# *Poria cocos* polysaccharide—functionalized graphene oxide nanosheet induces efficient cancer immunotherapy in mice

**DOI:** 10.3389/fbioe.2022.1050077

**Published:** 2023-01-16

**Authors:** Jinning Yang, Xiaoxiao Dong, Boye Li, Tian Chen, Boyang Yu, Xiaoli Wang, Xiangnan Dou, Bo Peng, Qin Hu

**Affiliations:** ^1^ The Faculty of Environment and Life, Beijing University of Technology, Beijing, China; ^2^ Beijing International Science and Technology Cooperation Base of Antivirus Drug, Beijing University of Technology, Beijing, China; ^3^ Institute of Medical Biotechnology, Chinese Academy of Medical Sciences, Beijing, China; ^4^ Civil Aviation Medicine Center, Civil Aviation Administration of China, Beijing, China; ^5^ Institute of Chinese Materia Medica, China Academy of Chinese Medical Sciences, Beijing, China

**Keywords:** nanovaccine, graphene oxide nanosheet, Poria cocos polysaccharides, cancer immunotherapy, vaccine delivery and adjuvant systems

## Abstract

**Introduction:** Tumor vaccines that induce robust humoral and cellular immune responses have attracted tremendous interest for cancer immunotherapy. Despite the tremendous potential of tumor vaccines as an effective approach for cancer treatment and prevention, a major challenge in achieving sustained antitumor immunity is inefficient antigen delivery to secondary lymphoid organs, even with adjuvant aid.

**Methods:** Herein, we present antigen/adjuvant integrated nanocomplexes termed nsGO/PCP/OVA by employing graphene oxide nanosheet (nsGO) as antigen nanocarriers loaded with model antigen ovalbumin (OVA) and adjuvant, *Poria cocos* polysaccharides (PCP). We evaluated the efficacy of nsGO/PCP/OVA in activating antigen-specific humoral as well as cellular immune responses and consequent tumor prevention and rejection *in vivo*.

**Results:** The optimally formed nsGO/PCP/OVA was approximately 120–150 nm in diameter with a uniform size distribution. Nanoparticles can be effectively engulfed by dendritic cells (DCs) through receptor-mediated endocytosis, induced the maturation of DCs and improved the delivery efficiency both *in vitro* and *in vivo.* The nsGO/PCP/OVA nanoparticles also induced a significant enhancement of OVA antigen-specific Th1 and Th2 immune responses *in vivo*. In addition, vaccination with nsGO/PCP/OVA not only significantly suppressed tumor growth in prophylactic treatments, but also achieved a therapeutic effect in inhibiting the growth of already-established tumors.

**Conclusion:** Therefore, this potent nanovaccine platform with nanocarrier nsGO and PCP as adjuvants provides a promising strategy for boosting anti-tumor immunity for cancer immunotherapy.

## Introduction

Recently, cancer immunotherapy has become one of the most promising techniques of both cancer prevention and intervention by virtue of its effective avoidance of off-target effects which can better improve anti-tumor immune responses (Arya et al., 2018). Among all kinds of cancer immunotherapy, the introduction of vaccines is rapidly becoming a growing trend in cancer treatment (Bachmann and Jennings, 2010). Recent studies have focused on different types of cancer vaccines, including tumor cell lysates, dendritic cells (DCs), nucleic acids (such as mRNA), and neoantigens (Bao et al., 2011). However, antigens alone are poor activators of adaptive immune responses. In the absence of adjuvants, antigens targeting immature DCs without inflammation or any microbial stimulation induce tolerance instead of effective immune responses. Recently, a number of adjuvants, including MF59, CpG ODN, AS04, and AS01, have been approved for use in human vaccines; however, the systemic immune toxicity induced by adjuvants continues to be an obstacle for widespread applications (Didierlaurent et al., 2017; Wilkins et al., 2017). Thus, innovative adjuvants with fewer adverse effects and greater modifiability are urgently required.

*Poria cocos* is an edible mushroom and has been used for medicine for a long history owing to its specific characterization and biological activities (Zhu et al., 2019; Liang et al., 2022; Zheng et al., 2022). *Poria cocos* is composed of multiple chemical composition, including triterpenes, polysaccharides, steroids, amino acids, choline, and histidine. Previous studies have revealed that *Poria cocos* polysaccharides (PCPs) and their derivatives have various biological activities, such as anti-tumor, anti-inflammatory, anti-viral, and anti-oxidant (Chen et al., 2009; Ke et al., 2010; Xu et al., 2022). The capability of PCP to enhance cellular immunity and humoral immunity and its outstanding safety profile make it a promising candidate for innovative adjuvant development (Zhang et al., 2019). However, even with adjuvants, inefficient delivery of antigens and adjuvants to secondary lymphoid organs often results in poor immune responses (Arya et al., 2018). Thus, an adjuvant can be co-formulated with an antigen within the same delivery carrier to induce effective immune responses.

Nanoparticles are minute particles that are typically <200 nm in diameter (Chrastina et al., 2011) and exhibit unique properties, such as large surface-to-volume ratio, drug-loading compacity and tunable surface chemistry, confer many advantages in terms of vaccine delivery (Xu et al., 2015). Recently, graphene oxide (GO) have been widely utilized as vehicles for drug delivery (Zheng et al., 2018), biological sensors (Xu et al., 2022) and have been applied successfully in photodynamic therapy (Wei et al., 2016), cancer treatment (Arya et al., 2018) and antibacterial therapy (Wang et al., 2022). GO is derived from graphite *via* a variety of oxidation processes, the most common of which is the enhanced Hummers method (Zhang et al., 2022). Because of its oxygen-containing functional groups, aromatic lattice, and large interfacial surface area, GO has the flexibility and capacity to load a wide range of compounds including drugs, nucleic acids, and peptides by non-covalent interactions (hydrophobic interactions, hydrogen bonding, and π–π stacking). In recent decades, there has been an increasing amount of research regarding the capacity of GO for the encapsulation and delivery of antigens. According to Dudek et al. (2016) antigen-loaded GO stimulated the immune system by up-regulating inflammatory cytokines, inducing lymphocyte proliferation and differentiation, and thus aided in the elimination of intracellular pathogens and infected cells after immunization. GO nanosheets (nsGOs) are derived from GO and have a small size (−100 nm) and tight size distribution. nsGO exhibits effective cell membrane permeability and low cytotoxicity (Wang et al., 2014). Thus, nsGO can be used as a potential carrier platform for antigen and adjuvant co-delivery.

In view of these considerations, we generated a safe and effective nsGO nanovaccine that could co-deliver the model antigen ovalbumin (OVA) protein and adjuvant PCP to induce robust immune responses and antitumor effects in a tumor-bearing mouse model. The formed nsGO/PCP/OVA nanoparticles induced DC maturation without detectable cytotoxicity and promoted antigen uptake both *in vitro* and *in vivo*. In addition, the nsGO/PCP/OVA nanoparticles provoked strong Th1 and Th2 type immune responses in vaccinated mice and functioned as a prophylactic vaccine to protect mice from E.G7-OVA tumor challenge, as well as a therapeutic vaccine to achieve better anti-tumor effects. These data demonstrate that nsGO/PCP/OVA may be an effective approach for enhancing antigen-specific adaptive immune responses against cancer cells.

## Methods

### Materials

CpG ODN 1668 (TCCATGACGTTC CTGATGCT) with a single-stranded phosphonothioate was obtained from Sangon Biotech (Sangon Biotech, China). PCP was obtained from the YuanYe Company (YuanYe inc, China). OVA protein was purchased from Sigma (Sigma, MO, United States), OVA peptide 257–264 (SIINFEKL) was from the Chinese Peptide Company (Chinese Peptide Company, China), and Alexa Fluor™ 647 conjugated OVA was purchased from Thermo (Thermo Fisher Scientific, MA, United States). All materials used in vaccines were purified using Pierce™ High-Capacity Endotoxin Removal Spin Columns (Thermo Fisher Scientific, MA, United States). Afterwards, the endotoxin levels were measured to be constantly below 5 Endotoxin Unit (EU)/mL using the ToxinSensor™ Endpoint Chromosome Endotoxin Detection Kit (Genscript, NJ, United States).

### Cell culture

Mouse lymphoma cell line E.G7-OVA cell was obtained from the American Type Culture Collection (ATCC, CRL-2113, MD, United States) and cultured in RPMI 1640 medium (ATCC, Cat# 30-2001, MD, USA) at 37°C in a humidified atmosphere containing 5% CO_2_. Mouse dendritic cell line DC2.4 cells were purchased from Bena Culture Collection (BNCC Inc, China) and incubated in RPMI 1640 medium (Gibco, NY, United States) with 2 mM glutamine, streptomycin-penicillin solution, 50 μM 2-mercaptoethanol, and 10% heat-inactivated fetal calf serum (FBS, Gibco, NY, United States). Bone marrow-derived DCs (BMDCs) were obtained from female C57BL/6 mice. Briefly, fresh BMDCs were isolated from the femur and tibia of C57BL/6 mice. Cells were then cultured in RPMI 1640 medium (Gibco, NY, United States) with 20 ng/mL GM-CSF (PeproTech, NJ, United States), penicillin-streptomycin solution (Solarbio, China), 50 μM 2-mercaptoethanol (Invitrogen, CA, United States), and 10% FBS (Gibco, NY, United States). Non-adherent and loosely adherent cells were harvested on day 5 or day 6 as immature BMDCs.

### Animals

57BL/6 (6–8 weeks old) female mice were obtained from Vital River Laboratory Animal Technology Co., Ltd. (Vital River Laboratory Animal Technology Co., Ltd. China) and housed in a specific-pathogen-free (SPF) animal laboratory. All animals are free for sterile food and water. After the injection procedure, the animals were closely monitored for symptoms of food intake, pain or distress, and motility. Mice were euthanized by cervical dislocation at humane endpoints or at the end of the experiments. All animal experiments were reviewed and approved by the Institutional Animal Treatment and Use Committee of the China Academy of Chinese Medica Sciences (code:2021B218).

### Preparation of nsGO

GO was generously provided by Dr. Dongtang Zhang (Beijing University of Technology, China) and nsGO was prepared according to Ying’s protocol (Wang et al., 2014). Briefly, GO was dissolved in water at a concentration of 0.2 mg/mL and sonicated in water bath for 2 h. After sonication in an ice bath with a sonification power of 40 W, NaOH solution was added to reach a final concentration of 5 M NaOH. Then, the resultant solution was sonicated in water bath for 2 h, and the pH of the solution was adjusted to neutral. The solution was centrifuged at 1,6128 × *g* for 10 min, and the supernatant was designated as nsGO.

### Preparation and characterization of nsGO/PCP/OVA

Epichlorohydrin was added to the nsGO solution at 40°C for 4 h under N_2_ protection. Once the unreacted epichlorohydrin was removed by ultrafiltration, endotoxin-free PCP solution (10 mg/mL) was added to epichlorohydrin-GO solution in water bath at 42°C for 3 h. After the pH of the nsGO/PCP solution was adjusted to weak acidity, a 1-(3-dimethylaminopropyl)-3-ethylcarbodiimide hydro/N-hydroxy succinimide (EDC/NHS) mixture was added, followed by incubation for 30 min. Subsequently, the OVA protein solution (1 mg/mL) was added to the prepared solution for 1 h at room temperature. Then, the unreacted protein was removed by ultrafiltration and the lyophilized solid was collected as nsGO/PCP/OVA nanoparticles. The concentration of nsGO/PCP/OVA was quantified by the OVA level using the Bicinchoninic Acid Assay kit (BCA, Biomed Inc, CA, United States). The encapsulation efficiency was measured using the following formula: [(Total OVA–Free OVA)/Total OVA] ⋅ 100%. The nanoparticles were characterized using transmission electron microscopy (TEM, Hitachi, Japan), Fourier Transform infrared spectroscopy (FT-IR spectroscopy, Spectrum 100 FTIR, PerkinElmer, MA, USA), and dynamic light scattering (DLS, Malvern, Germany). TEM images were analyzed for the nanoparticle size distribution using Nano measurer 1.2 software (Fudan University, China).

### Cell viability

To evaluate the cytotoxic effects of nanoparticles, DC2.4 cells were incubated with nsGO/PCP/OVA ranging from 0 to 100 μg/mL OVA. The absorbance at 450 nm was measured using the Cell Counting Kit-8 (Dojindo, Japan) 24 h after treatment. The percentage of cell viability of DC2.4 cells was calculated.

### Flow cytometry analysis

For cell surface marker detecting, the cells were washed with PBS and blocked with anti-mouse CD16/32 antibody (BioLegend, CA, United States). After treatment for 15 min at room temperature, the cells were incubated with different antibodies in PBS with 1% FBS (Gibco, NY, United States) for 1 h. After washed 3 times with PBS, cells were and resuspended in PBS with 1% FBS. Flow cytometry analysis was performed using a BD FACS Calibur™ flow cytometer (BD Bioscience, CA, United States), and data were analyzed using FlowJo software V10 (Tree star, OR, United States).

### Bone marrow-derived DCs activation

Immature BMDCs were collected and treated with 20 μg/mL OVA, 5 μg/mL nsGO, 100 μg/mL PCP, nsGO/PCP, and nsGO/PCP/OVA, according to Dong’s research (Dong et al., 2021). After 24 h, the supernatants were collected and measured for IL-6 and IL12 production (ELISA MAX Deluxe kits, BioLegend, CA, United States). The expression of CD80, CD86, and MHC-II on BMDC cell surface was analyzed using PE anti-mouse MHC-II (BioLegend, CA, United States), APC anti-mouse CD80 (BioLegend, CA, United States), and PerCP/Cyanine5.5 anti-mouse CD86 (BioLegend, CA, United States) by FACS analysis.

### Uptake assays

For *in vitro* uptake assays, BMDCs were incubated with PBS, 5 μg/mL OVA-FITC, and 2.5, 5, and 10 μg/mL nsGO/PCP/OVA-FITC. Cells incubated with nsGO/PCP/OVA-FITC (5 μg/mL) at 4°C were used as controls. After 30 min of incubation, the BMDC cells were collected and stained with APC anti-mouse CD11c (BioLegend, CA, United States) antibody before FACS analysis. For uptake competition assays, cells were pre-treated with PCP, OVA, or 200 μg/mL mannans (Solarbio, China) for 30 min. BMDCs were then treated with nsGO/PCP/OVA-FITC (10 μg/mL) for 45 min. BMDCs were also incubated with nsGO/PCP/OVA (10 μg/mL) for 30 min, followed by co-culture with 100 μg/mL Lucifer yellow VS. dilithium salt (LY, Sigma, MO, United StatesA) for 45 min before FACS analysis. To monitor the *in vivo* uptake of nanoparticles, mice were injected with OVA-Alexa 647 (20 μg) or nsGO/PCP/OVA-Alexa 647 (containing 20 μg OVA) in both hind footpads. After 6 h, the popliteal lymph nodes were dissected and incubated with collagen D (Sigma, MO, United States) and DNase (Sigma-Aldrich, MO, United States) to prepare single-cell suspensions. Cells were incubated with anti-mouse CD16/32 antibody (BioLegend, CA, United States) for 15 min and then stained with FITC-anti-mouse CD11c antibody (BioLegend, CA, United States) before FACS analysis.

### Immunization of mice

Female C57BL/6 mice were randomly allocated to six groups. On days 0, 7, and 14, mice were immunized subcutaneously with PBS (control), OVA (20 μg per mouse), PCP + OVA (250 μg PCP and 20 μg OVA per mouse), CpG ODN + OVA (10 μg CpG and 20 μg OVA per mouse), nsGO/PCP + OVA (20 μg OVA per mouse), and nsGO/PCP/OVA (containing 20 μg OVA per mouse). On day 21, the mice were anesthetized with isoflurane and sacrificed following the collection of serum and spleen for subsequent analysis.

### Measurement of Th1 and Th2 immune responses

Splenocytes were harvested from mice on day 21 after the three vaccinations. For ELISPOT, cells were cultured at 2 × 10^5^ cells/well in ELISPOT plates (Dakewe, China), and stimulated with 1 μg/mL OVA peptide_257-264_ (SIINFEKL, OVA Ⅰ) or 300 μg/mL OVA protein for 36 h. The number of spots was counted, and the results were expressed as spot-forming cells (SFCs) per 10^5^ splenocytes. For the cell proliferation assay, splenocytes were cultured at 2 × 10^6^ cells/mL with 300 μg/mL OVA 300 μg/mL or 1 μg/mL OVA Ⅰ 1 μg/mL. After 48 h, cells were collected, incubated with anti-mouse CD16/32 antibody, and stained with anti-mouse CD69, anti-mouse CD3 (Biolegend, CA, United States), anti-mouse CD4 (Biolegend, CA, United States), and anti-mouse CD8 (Biolegend, CA, United States) antibodies at room temperature for 30 min. To measure cytokine release, splenocytes were cultured with OVA (300 μg/mL) or OVA I (1 μg/mL) for 72 h, supernatants were collected and measured for IL-4 and IFN-γ concentrations using ELISA kits (BioLegend, CA, United States).

### Prophylactic tumor challenge assays

For prophylactic vaccination, C57BL/6 mice were first primed with different formulations on days −21 and boosted on days −14 and −7 as above. On day 0, mice were subcutaneously injected with 2 × 10^5^ E.G7-OVA cells in the right flank. On day 21, all mice were euthanized when a humane endpoint was reached, tumor masses were measured, and tumor volume was calculated as length × width^2^ × 0.5. Blood samples were collected for antibody detection.

### Therapeutic tumor vaccination

C57BL/6 mice were subcutaneously injected with 2 × 10^5^ E.G7-OVA cells into the right flank. When the mean tumor size reached approximately 100 mm^3^, mice were inoculated with different formulations. All mice were euthanized on day 18, when the control mice reached the humane endpoint. Blood and spleen tissues were collected for further analysis.

### ELISA

For antibody detection in prophylactic tumor assays, serum was collected from mice on day 21. The antibody titers of serum OVA-specific IgG1 and IgG2a were measured using an IgG Mouse ELISA kit (Thermo Fisher Scientific, MA, United States). In therapeutic tumor assay, to detect anti-OVA specific antibodies, serum was collected on day 18 and diluted (1:1,000 to detect IgG, 1:500 to detect IgG1, and 1:3,000 for IgG2a) for serum OVA-specific IgG, IgG1, and IgG2a measurements using ELISA kit (Thermo Fisher Scientific, MA, United States).

For cytokine measurements in tumor-bearing mice, splenocytes were collected and cultured at 2 × 10^6^ cells/mL with 300 μg/mL OVA. After 72 h, the supernatant of cell culture was collected and IL-2 and IL-4 concentrations were measured using ELISA kits (BioLegend, CA, United States).

### Statistical analyses

Statistical analyses were performed using Prism 7.0 (GraphPad Software Inc, CA, United States). All data analysis results in this study are expressed as means ± SD and were analyzed by one-way analysis of variance (ANOVA). *p* < 0.05 was defined as statistical significance.

## Result

### Preparation and characterization of nsGO/PCP/OVA

**FIGURE 1:**
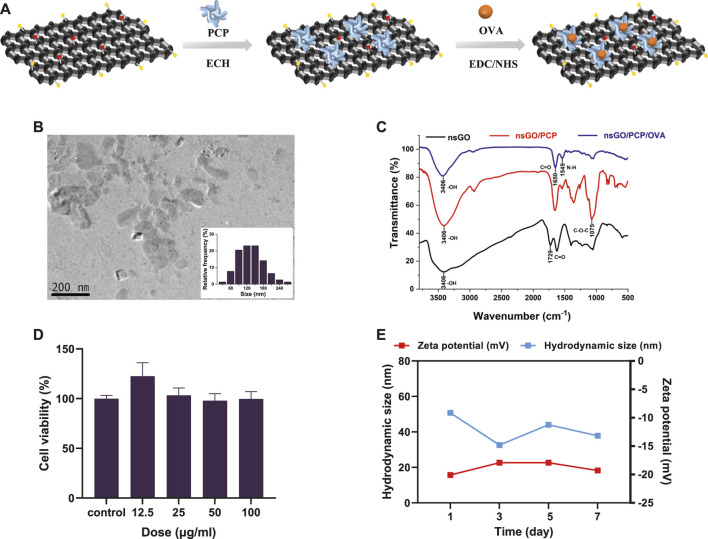
Preparation and characterization of nsGO/PCP/OVA. **(A)** Schematic representation for the preparation of nsGO/PCP/OVA. **(B)** TEM image and size distribution of nsGO/PCP/OVA. The inset in **(B)** is the size of nsGO/PCP/OVA nanoparticle. **(C)** FT-IR spectra of nsGO, nsGO/PCP and nsGO/PCP/OVA. **(D)** ζ potentials and size distribution of nsGO/PCP/OVA for a week. **(E)** Viability of DC2.4 cells after nsGO/PCP/OVA treatment was evaluated by CCK-8 assay. Data shown are representative of 3 replicate experiments.

The synthetic route to nsGO/PCP/OVA is illustrated in Figure 1A. nsGO was first conjugated with PCP and then with OVA, and the OVA antigen encapsulation efficiency was calculated as 43.5 ± 4.5%. TEM revealed that nsGO/PCP/OVA was well-dispersed and exhibited a size of approximately 120–150 nm (Figure 1B). The combination of PCP and OVA with nsGO was also confirmed by FT-IR spectroscopy (Figure 1C), and a broad band at 3,406 cm^−1^, attributed to OH groups, and bands at 1,729 cm^−1^, typical of carbonyl groups, were observed in GO nanosheets. The presence of a C-O-C stretching peak at 1,075 cm^−1^ indicated that the polysaccharide was inserted into the nsGO. Two distinct amide I and II peaks for the protein were observed at 1,650 cm^−1^ and 1,545 cm^−1^. The ζ potentials and size distribution of nsGO/PCP/OVA were monitored by DLS analysis for 7 days, and the constructed nanoparticles exhibited sustained storage stability (Figure 1D). These data suggested that PCP and OVA successfully bonded to the GO nanosheets. We also evaluated the cytotoxicity of nsGO/PCP/OVA on DC2.4 cells (Figure 1E) as well as nsGO (Supplementary Figure S1) and nsGO/PCP/OVA on BMDCs (Supplementary Figure S2). As revealed by CCK-8 assays, DC2.4 cells treated with 0–100 μg/mL nsGO/PCP/OVA showed no obvious cytotoxicity, indicating no detectable cytotoxic effect of nsGO/PCP/OVA *in vitro* at a concentration of 100 μg/mL.

### nsGO/PCP/OVA induced maturation of BMDCs and enhanced OVA uptake

**FIGURE 2:**
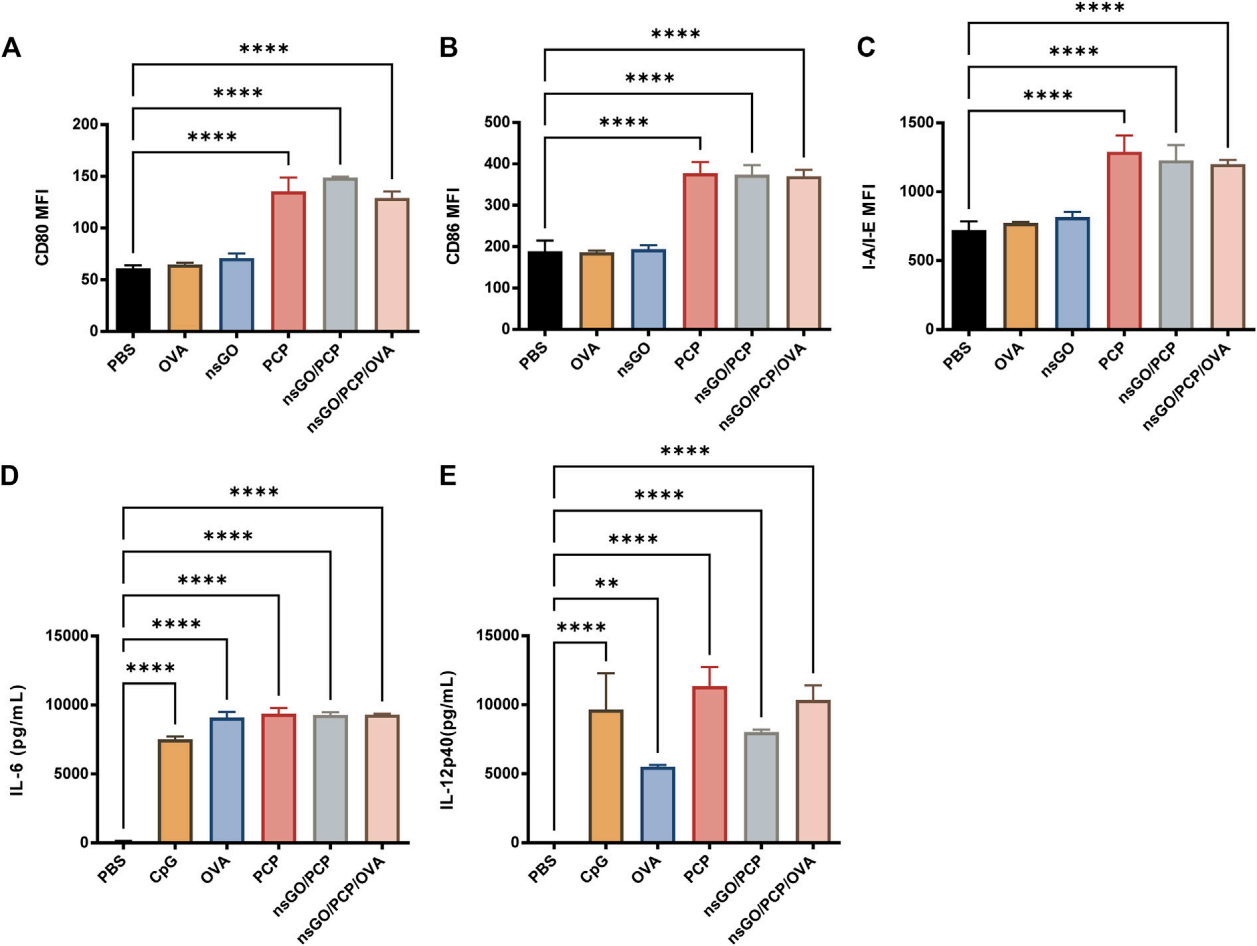
*In vitro* BMDC maturation induced by nsGO/PCP/OVA. Immature BMDCs were isolated from C57BL/6 female mice and stimulated with 20 μg/mL OVA, 5 μg/mL nsGO, 100 μg/mL PCP, nsGO/PCP, and nsGO/PCP/OVA for 24 h. Expression of BMDC surface markers CD80 **(A)**, CD86 **(B)**, and I-A/I-E **(C)** were analyzed by flow cytometry. Supernatant of BMDC culture was collected, IL-6 **(D)** and IL-12 **(E)** secretion by BMDCs was measured by ELISA. Values presented are the means ± SD of three replicates, **, *p* < 0.01; ****, *p* < 0.0001 vs. PBS control.

nsGO/PCP/OVA-induced maturation of DCs was assessed by measuring co-stimulatory factor expression and cytokine release by BMDCs. Compared to the control, nsGO/PCP and nsGO/PCP/OVA induced a 2-3-fold upregulation in the surface expression of CD80 (Figure 2A), CD86 (Figure 2B), and MHC II (referred to as I-A/I-E, Figure 2C). In addition, nsGO/PCP/OVA-treated DCs exhibited significantly higher production of interleukin 6 (IL-6) and interleukin 12 (IL-12) as determined by ELISA (Figures 2D,E). These data suggested that nsGO/PCP/OVA significantly induced BMDC maturation.

**FIGURE 3:**
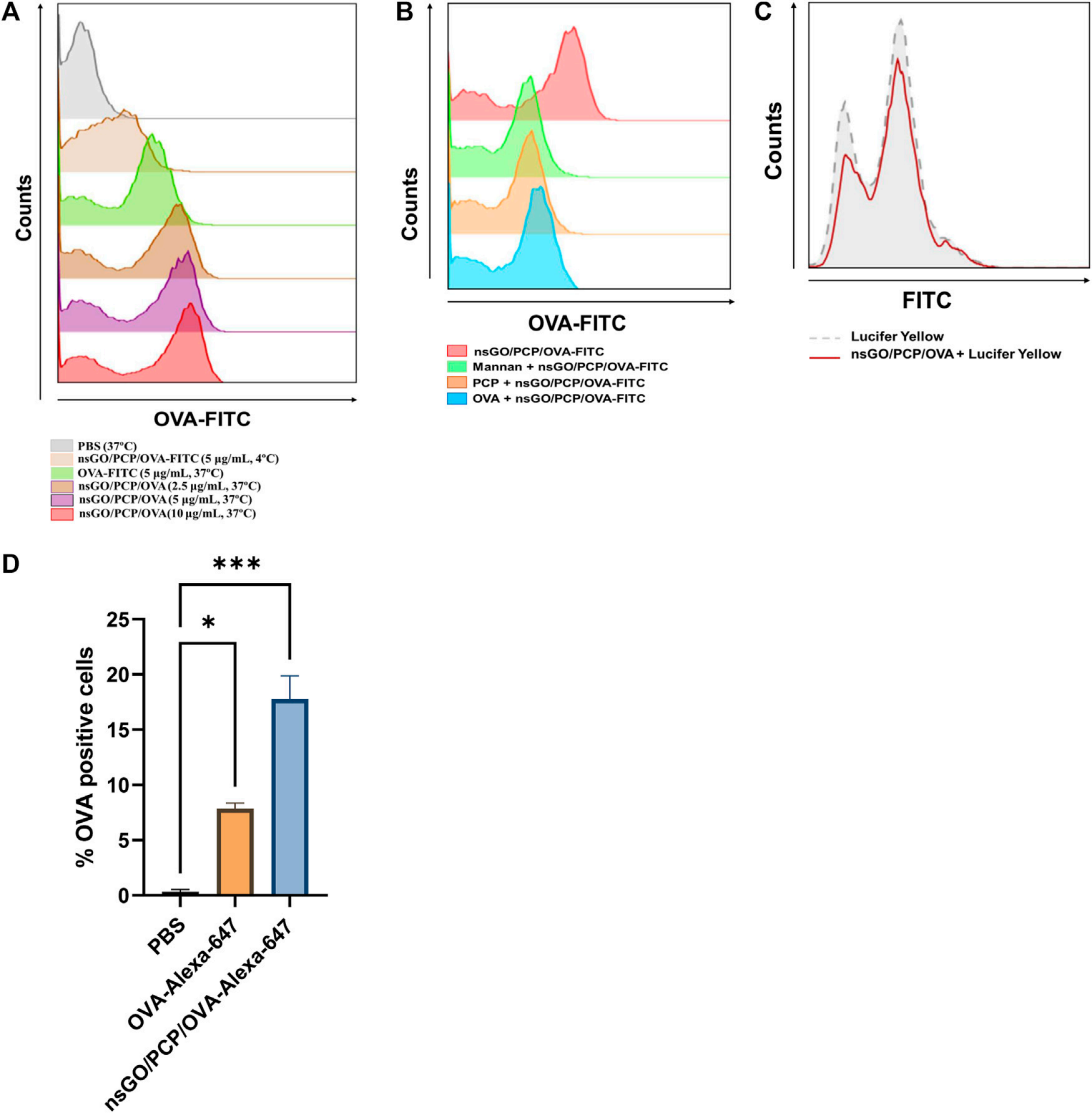
Uptake of nsGO/PCP/OVA *in vitro* and *in vivo*. **(A)** BMDCs were pre-incubated with PBS, OVA-FITC at 5 μg/mL, nsGO/PCP/OVA-FITC at 2.5, 5, 10 μg/mL at 37°C or nsGO/PCP/OVA-FITC at 5 μg/mL at 4°C for 30 min, OVA-FITC-positive BMDCs were detected by FACS analysis. **(B)** BMDCs were pre-incubated with PCP, OVA or mannan for 30 min, cells were cultured with nsGO/PCP/OVA-FITC (10 μg/mL) for 45 min in 37°C before FACS analysis. **(C)** BMDCs were first incubated with nsGO/PCP/OVA-FITC (10 μg/mL) for 30 min, then BMDCs were co-cultured with Lucifer Yellow for 45 min at 37°C before analysis of antigen uptake by CD11c^+^ BMDCs. The uptake of Lucifer Yellow is shown in histograms (Grey area: cells without pre-incubation with nsGO/PCP/OVA, red line: cells incubated with nsGO/PCP/OVA). **(D)** Mice were injected with PBS, OVA-Alexa 647 (20 μg per mouse) and nsGO/PCP/OVA-Alexa 647 (20 μg OVA per mouse) in both footpads. After 6 h, popliteal lymph nodes were isolated and prepare for single-cell suspension. Cells were then stained with anti-mouse CD11c antibody to analyze Alexa 647-positive cells using FACS analysis. Values presented are the means ± SD of three replicates (n = 3), *, *p* < 0.05; ***, *p* < 0.001 vs. PBS control.

The cellular uptake of FITC-labeled nsGO/PCP/OVA by DCs was measured using flow cytometry. According to Figure 3A, there is a dose-dependent increase in uptake of nsGO/PCP/OVA nanoparticles by DCs compared to free OVA and nsGO/PCP/OVA at 4°C. To get a better understanding of the uptake route of nsGO/PCP/OVA, BMDCs were pre-incubated with free PCP, free OVA, and mannan, which was used to block the mannose receptor, a well-recognized endocytic receptor responsible for OVA uptake by BMDCs. The results showed that free PCP, OVA, and mannan caused a decrease in the percentage of OVA-FITC positive cells, indicating that PCP and OVA could both mediate the uptake of nsGO/PCP/OVA, and multiple receptors, including the mannose receptor, were involved in the receptor-mediated endocytosis of nsGO/PCP/OVA (Figure 3B). In addition, we incubated BMDCs with Lucifer Yellow, a well-known marker for pinocytosis. As depicted in Figure 3C, there is no reduction in the uptake of Lucifer Yellow, suggesting that the nanoparticles interfered with receptor-mediated endocytosis, but not pinocytosis. Moreover, the efficiency of nanovaccines in co-delivering antigens and adjuvants to lymph nodes was assessed *in vivo*. After injection of Alexa 647-labeled nsGO/PCP/OVA, the draining lymph nodes of mice were collected, and CD11c^+^ DCs were prepared for analyzing OVA-Alexa 647 positive cells. As shown in Figure 3D, nsGO/PCP/OVA significantly enhanced the uptake of OVA by DCs in draining lymph nodes, compared to OVA alone. These data suggested that nsGO/PCP/OVA promoted the uptake of antigens both *in vitro* and *in vivo*.

### nsGO/PCP/OVA induced Th1 and Th2 immune responses *in vivo*

**FIGURE 4:**
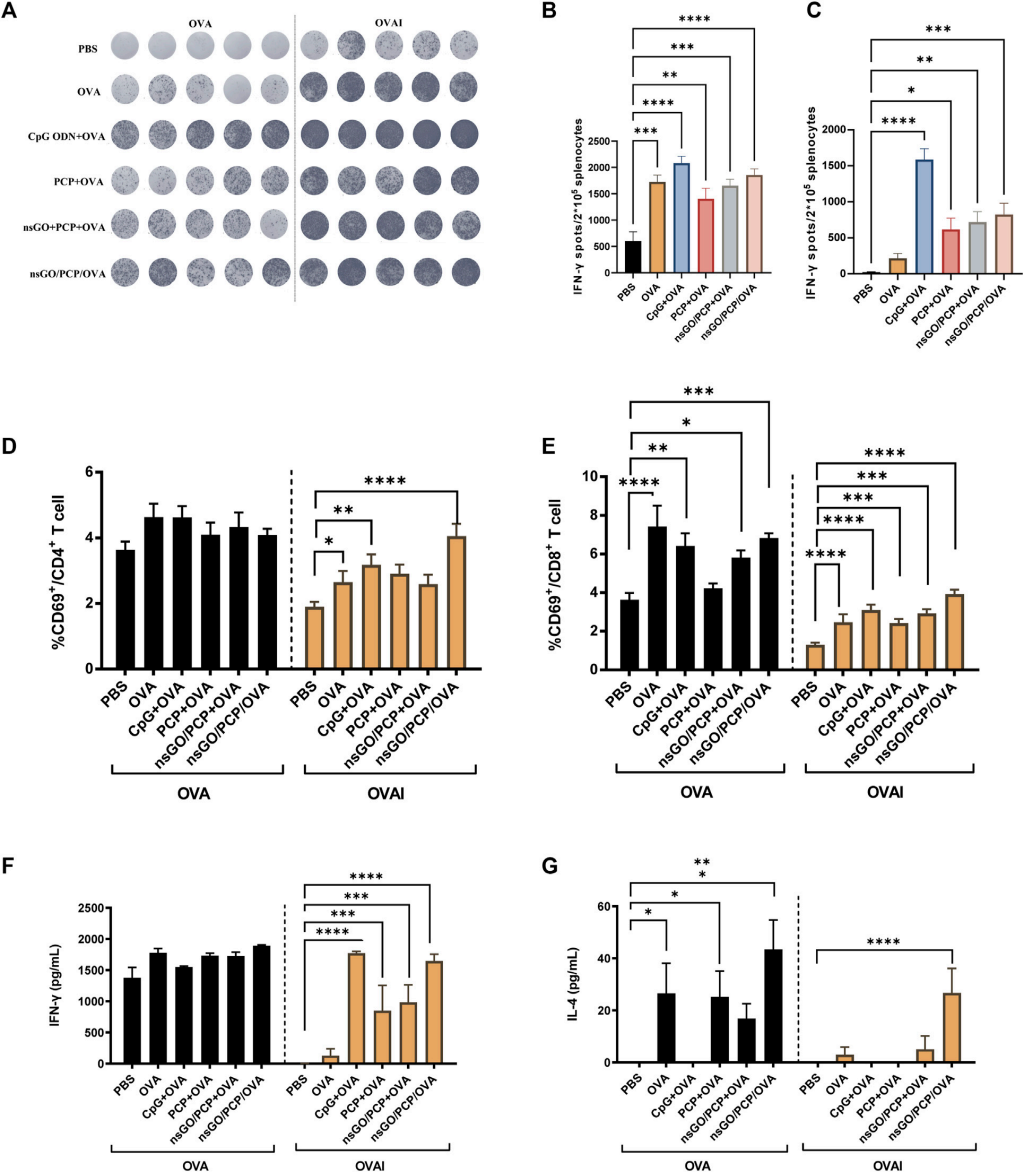
Stimulation of T cells in immunized mice by nsGO/PCP/OVA. Mice were immunized 3 times (s.c.) with PBS, OVA, PCP + OVA, CpG ODN + OVA, nsGO/PCP + OVA or nsGO/PCP/OVA at 1 week intervals. Blood and spleen tissues were collected on day 21. **(A)** Splenocytes were stimulated with 300 μg/mL OVA protein or 1 μg/mL OVA peptide_257–264_ (OVAI) per well. After 36 h, IFN-γ secreting cells were visualized and analyzed by ELISPOT. The number of OVA-stimulated **(B)** and OVA Ⅰ- **(C)** stimulated IFN-γ secreting cells was measured by ELISPOT. Splenocytes were co-cultured with OVA (300 μg/mL) or OVA Ⅰ (1 μg/mL) for 48 h, CD69^+^ CD4^+^ T **(D)** and CD69^+^ CD8^+^ T **(E)** cells were analyzed using FACS. Supernatants of splenocytes were collected at 72 h and measured for IFN-γ **(F)** and IL-4 **(G)** levels using ELISA kits. Data are presented as means ± SD (n = 5), *, *p* < 0.05; **, *p* < 0.01; ***, *p* < 0.001; ****, *p* < 0.0001 vs. PBS control.

After three vaccinations with nsGO/PCP/OVA at 1 week intervals, mouse spleens were collected and assayed for the number of IFN-γ-producing cells, CD69 expression, as well as cytokine production. After stimulation with OVA or OVA I for 36 h, IFN-γ-secreting cells were quantitated by ELISPOT (Figure 4A), which showed that both OVA-specific (Figure 4B) and OVA I-specific (Figure 4C) IFN-γ-secreting CD8^+^ T cell numbers significantly increased after nsGO/PCP/OVA vaccination, suggesting that nsGO/PCP/OVA activated OVA-specific CD4^+^ and CD8^+^ T cells. The proliferation of OVA-specific CD8^+^ T cells after nsGO/PCP/OVA treatment was further supported by upregulation of CD69 expression in CD8^+^T cells (Figures 4D,E). Moreover, immunization with nsGO/PCP/OVA elevated the secretion of IFN-γ (Figure 4F) and IL-4 (Figure 4G) compared to the control group. Together, these results demonstrated the robust adjuvant effect of nsGO/PCP/OVA with enhanced Th1 and Th2 immune responses.

### Prophylactic and therapeutic effects of nsGO/PCP/OVA on E.G7-OVA tumor-bearing mice

**FIGURE 5:**
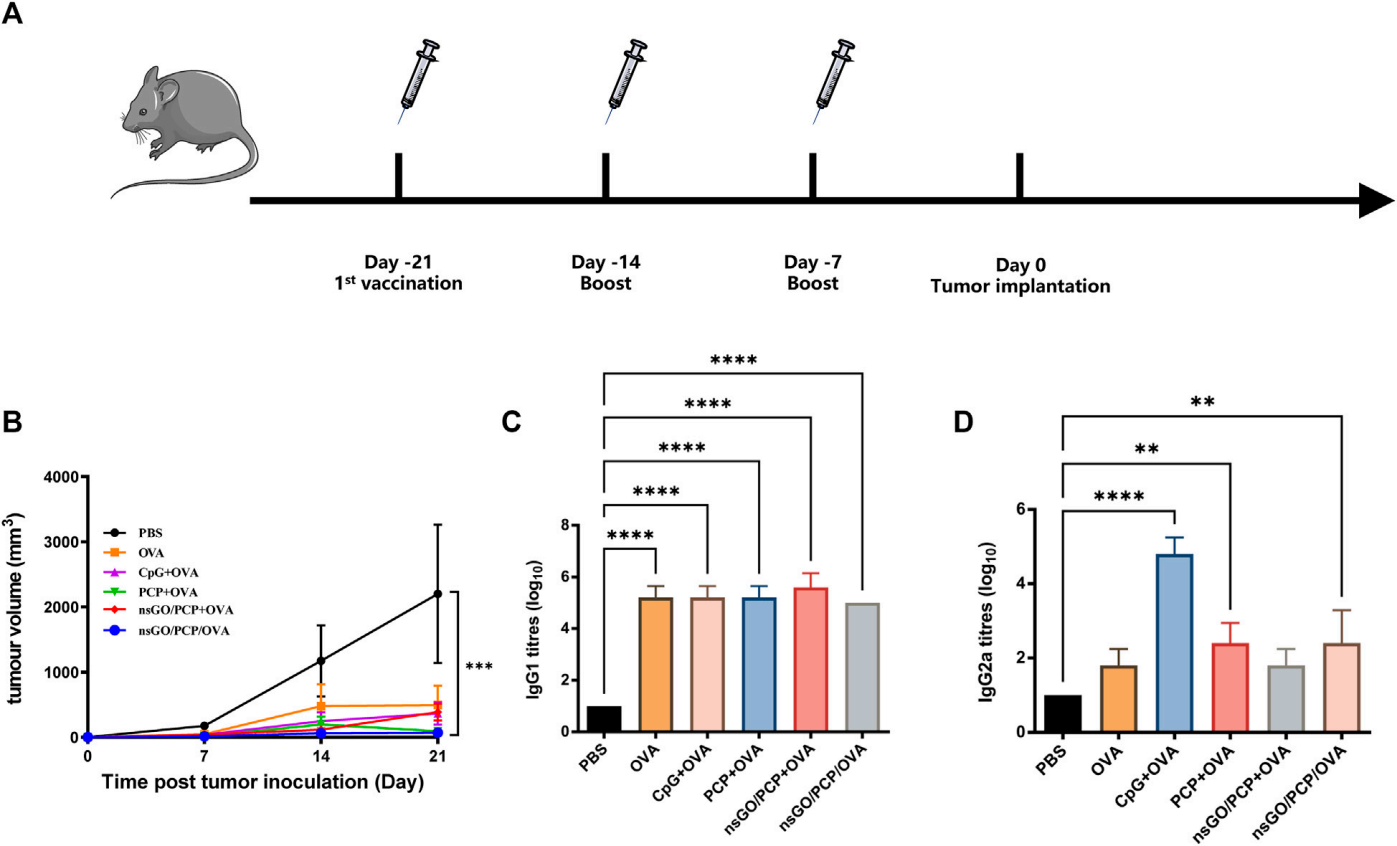
Prophylactic anti-tumor effects of nsGO/PCP/OVA on mice. **(A)** Immunization scheme for tumor prevention. Mice were primed subcutaneously with either PBS, OVA, CpG + OVA, PCP + OVA, nsGO/PCP + OVA, or nsGO/PCP/OVA and boosted with the same antigens twice at 1 week intervals. On day 0, 2 × 10^5^ **(E)**G7-OVA cells were injected subcutaneously into the right flank of mice. **(B)** On day 21, mice were sacrificed and tumor masses were measured. Blood was collected on day 21. The concentrations of OVA-specific IgG1 **(C)** and IgG2a **(D)** secretions in mouse serum were measured by ELISA. Data are presented as means ± SD (n = 5), **, *p* < 0.01; ****, *p* < 0.0001 vs. PBS control.

The prophylactic anti-tumor effect against E.G7-OVA tumor cells, a lymphoma cell line that stably expresses OVA, was evaluated to assess the potential of nsGO/PCP/OVA as an antineoplastic vaccine. C57BL/6 female mice were subcutaneously immunized with nsGO/PCP/OVA or control formulations 3 times at 1week intervals. As depicted in Figure 5A, all mice were challenged with E.G7-OVA cells 1 week after the final vaccination. As shown in Figure 5B, tumor growth in the nsGO/PCP/OVA group was significantly lower than that in the control group. In addition, data concerning serum OVA-specific antibody production showed that nsGO/PCP/OVA upregulated anti-OVA IgG1 (Figure 5C) and IgG2a (Figure 5D) antibodies, indicating that nsGO/PCP/OVA induced a potent immune response in tumor prophylaxis.

**FIGURE 6:**
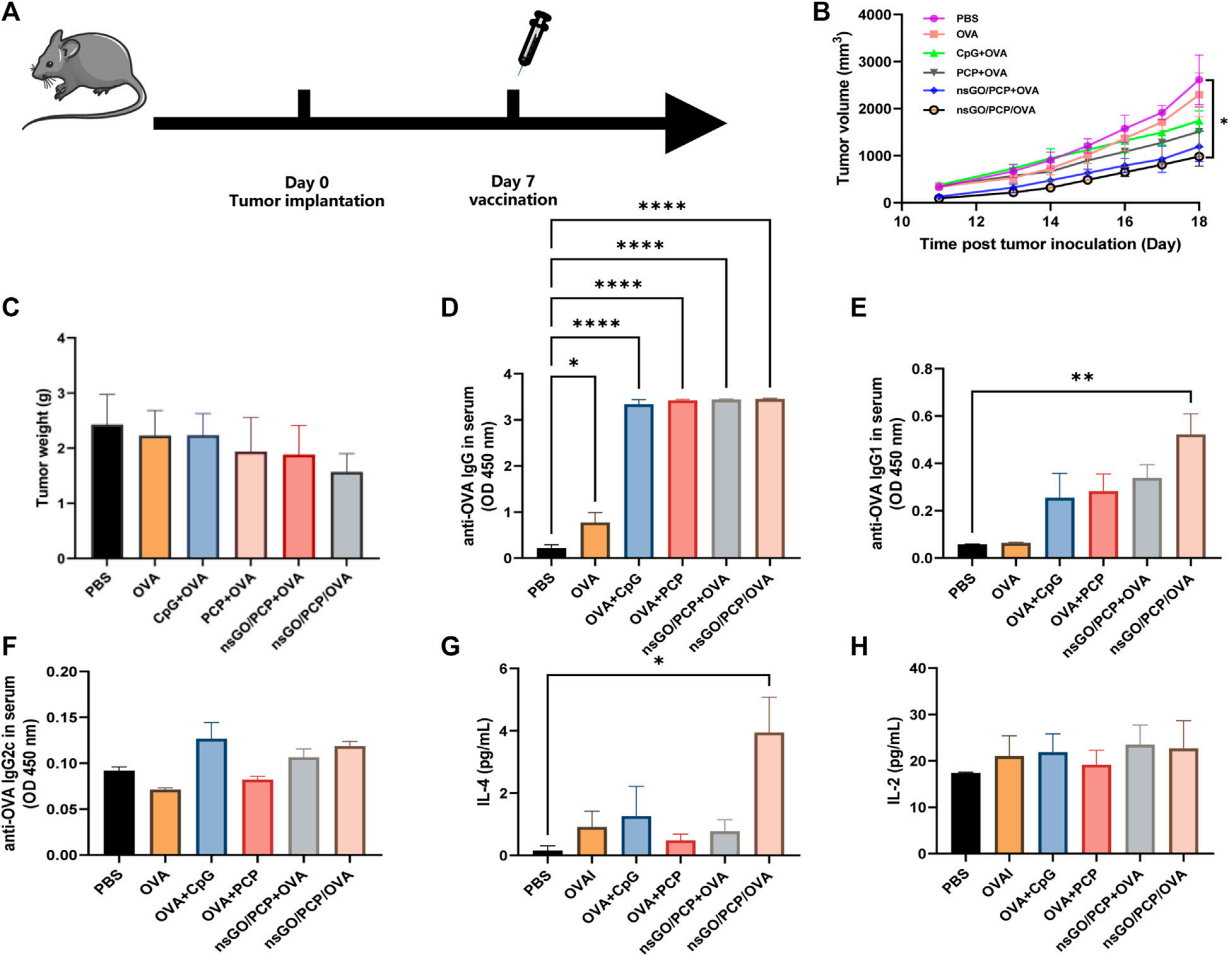
Therapeutic anti-tumor effect of nsGO/PCP/OVA on tumor-bearing mice. **(A)** The scheme of vaccines for tumor treatment. Mice were subcutaneously injected with 2 × 10^5^ **(E)** G7-OVA cells into the right flank. Seven days later, the mice were administered either PBS, OVA, CpG + OVA, PCP + OVA, nsGO/PCP + OVA, or nsGO/PCP/OVA. Tumor volumes **(B)** and weight **(C)** were measured after sacrifice on day 18 because the control mice reached a humane endpoint. Blood was collected on day 18 from tumor-bearing mice. The concentrations of OVA-specific IgG **(D)**, IgG1 **(E)** and IgG2a **(F)** secretions in mouse serum were measured by ELISA. Splenocytes were collected on day 18 and cultured with OVA (300 μg/mL) for 72 h, IL-4 **(G)** and IL-2 **(H)** concentrations in the supernatants of splenocytes were measured using ELISA kits. Data are presented as means ± SD (n = 5), *, *p* < 0.05; **, *p* < 0.01; ****, *p* < 0.0001 vs. PBS control.

Next, we evaluated the elimination of established tumors by inoculation with nsGO/PCP/OVA. One week after inoculation with E.G7-OVA tumor cells, mice received vaccination of nsGO/PCP/OVA and control formulas, respectively (Figure 6A). All mice were euthanized on day 18, when the control mice reached the humane endpoint, and a decline in tumor volume (Figure 6B) and weight (Figure 6C) was observed in the nsGO/PCP/OVA-treated mice. In addition, we analyzed OVA-specific anti-OVA IgG (Figure 6D), IgG1 (Figure 6E), IgG2c (Figure 6F) in serum and cytokine IL-4 (Figure 6G), IL-2 (Figure 6H) release from splenocytes. We found that nsGO/PCP/OVA significantly upregulated serum anti-OVA IgG (Figure 6D), IgG1 (Figure 6E) antibodies, and IL-4 release (Figure 6G), indicating that nsGO/PCP/OVA induced a potent humoral immune response in mice with tumors.

## Discussion

As an adjuvant, PCP is known for its potential to improve immunogenicity by triggering antigen-specific immune responses against cancer metastasis (Wu et al., 2016; Gai et al., 2017; Liu et al., 2020). However, owing to its relatively poor stability and untargeted features (Zhao et al., 2022), PCP is limited in clinical applications. Recently, the construction of nanoparticles has been shown to reduce drug loss during delivery, enhance the solubility of hydrophobic drugs, improve drug targeting, and extend drug release (Dudek et al., 2016; Wen et al., 2019). In the present study, we generated nanoparticles assembled from PCP adjuvant, OVA antigen, and nsGO nanosheets, and investigated their potential to enhance humoral and cellular immune responses and their therapeutic and prophylactic antitumor effects in E.G7-OVA-bearing mice. Our data demonstrated that nsGO/PCP/OVA induced robust activation of BMDCs and enhanced antigen uptake both *in vitro* and *in vivo*. Compared to free PCP, nsGO/PCP/OVA elicited stronger Th1 and Th2 responses in mice, as shown by the significant upregulation of IFN-γ-secreting CD8^+^ and CD4^+^ T cells, as well as the production of IFN-γ and IL-4. Furthermore, nsGO/PCP/OVA treatment exhibited antitumor effects against E.G7-OVA in both prophylactic and therapeutic mouse models.

The application of nanotechnology in drug delivery marks an unparalleled opportunity to change the foreseeable future of the pharmaceutical and biotechnological industries. GO offers excellent opportunities for; vaccination, such as enhancing antigen uptake by DCs and stimulating antigen-specific humoral and cellular immunity, thereby achieving robust cancer immunotherapy (Wang et al., 2022). GO has a typical two-dimensional crystal structure with a single atomic layer and oxygen functional groups. The basic skeleton of this 2D atomic planar structure is composed of crumpled sheets of sp^2–^ and sp^3–^ hybridized carbon atoms arranged in a hexagonal grid, which provides GO with hydrophobic nature and a large surface area. Hydrophilic groups including epoxy, carboxyl, and hydroxyl are dispersed over the basal planes and edges of the skeleton (Bao et al., 2011; Sharma et al., 2018). GO exhibit a large surface area that is almost 10 times the size of other nanomaterials (Bao et al., 2011), which endows GO with superiority on delivery over other materials. Owing to its unique physicochemical properties, GO has the potential to boost the immune system and thus could be employed to deliver antigens into DCs (Zhang et al., 2022). However, the toxic effects of GO on living cells and organs limit its application in the medical field. Researchers have demonstrated that Graphene-Family Nanomaterials (GFNs) can be toxic to cells; in particular, GFNs cause dose-dependent oxidative stress in cells owing to their inherent chemical properties (oxidation state and lateral size), and it is speculated that the biological response can be different over the material family depending on the number of layers, stiffness, lateral size, surface functionalization, hydrophobicity, and dose (Zhang et al., 2010; Chang et al., 2011; Wang et al., 2011; Sanchez et al., 2012; Jia et al., 2019). Previous reports have noted that reduced GO (rGO) and carboxylated graphene exhibited lower toxicity than GO or native graphene, indicating that the surface modification of graphene can affect its toxicity (Yang et al., 2010; Sasidharan et al., 2011). The functional derivatives of GO possess distinctive features that make it more effective for biomedical applications. Functionalized GO is distinguished by its ability to disperse in numerous solvents, which facilitates its use and lowers toxicity (Paredes et al., 2008). Therefore, in our study, we utilized nsGO with functional modifications to co-deliver antigens and adjuvants effectively with fewer side effects compared to GO, and no cytotoxicity of nsGO/PCP/OVA was detected in cell viability assays at a concentration of 100 μg/mL, further suggesting the safety of the formed nanoparticles.

Among all types of immunotherapies, the efficiency of delivering the antigen peptide and adjuvant to lymph nodes and engulfment by APCs is key factors affecting immunotherapeutic effects. Prior studies have noted the importance of particle size in determining vaccine access to lymph nodes (Zhang et al., 2017). Generally, nanoparticles with a size of 10–200 nm can enter lymphatic vessels and be effectively engulfed by APCs in the lymph nodes (Bachmann and Jennings, 2010). In this study, the size of the formed nsGO/PCP/OVA was stable at approximately 120–150 nm, which suggests its potential lymph node targeting capability. In addition, results from *in vivo* uptake assays further demonstrated the superiority of nanovaccines in delivering antigens and adjuvants to draining lymph nodes. The results from the *in vitro* study also confirmed that nsGO/PCP/OVA could effectively co-deliver PCP and antigenic peptide-OVA into DCs, as shown by the maturation and activation of DCs.

Sufficient expression of antigen and co-stimulatory molecules as well as the secretion of IL-6 and IL-12 are all necessary for effective DC function (Trinchieri, 2003; Kaka et al., 2008). According to our findings, nsGO/PCP/OVA induced DC maturation by enhancing the expression of MHC class II and co-stimulatory molecule, as well as cytokine secretion. To explore how nsGO/PCP/OVA entered DCs, we conducted a compete uptake assay by adding sufficient free PCP, free OVA, and mannan. Previous reports have noted that mannan is broadly utilized in the blockage of mannose receptors on DCs and the mannose receptor-mediated pathway has been reported to be involved in the uptake of OVA by DCs (Becker et al., 2006; Burgdorf et al., 2007). The data showed that mannan, PCP, and OVA significantly inhibited the uptake of nsGO/PCP/OVA, indicating that PCP and OVA mediated the uptake of nanoparticles, and that the mannose receptor was involved in receptor-mediated endocytosis. Furthermore, no significant reduction was found in Lucifer Yellow uptake, indicating that nsGO/PCP/OVA primarily affected receptor-mediated endocytosis rather than pinocytosis.

To further elucidate the capability of nsGO/PCP/OVA to enhance cellular immune responses, OVA was utilized as a model antigen, whose MHC I epitope (OVA 257–264) and MHC II epitope (OVA 323–339) have been well studied (Rötzschke et al., 1991; Lipford et al., 1993). We also used CpG ODN 1668 as a positive control to obtain a better understanding of the cellular immune response induced by nsGO/PCP/OVA. The TLR9 ligand CpG ODN, a well-documented Th1-related adjuvant, has been authorized for the use in the Heplisav-B (HepB-CpG) vaccine to aids cross-presentation of MHC I-restricted antigens since 2018 (Schillie et al., 2018; Hyer and Janssen, 2019). After three vaccinations, splenocytes of immunized mice were harvested and re-stimulated with OVA or OVA I. Successful antitumor vaccines likely require both CD 4^+^ and CD8^+^ T cell responses, as reported (Joffre et al., 2012). Effective cross-presentation of antigens by DCs plays crucial role of initiating optimal CD8^+^ T cell responses, especially in vaccines against intracellular antigens (intracellular microbes, viruses) and cancer. If nsGO/PCP/OVA could promote the cross-presentation of exogenous OVA protein in DCs, CD8^+^ T lymphocytes that particularly identify the SIINFEKL (MHC class I-restricted) epitope of OVA would proliferate and be capable of increasing IFN-γ secretion. This hypothesis was confirmed by FACS analysis, ELISPOT assays, and ELISA results in that nsGO/PCP/OVA were potent at cross-priming, activating specific cytotoxic CD8^+^ T cells, increasing the production of Th1 type cytokines as well, and triggering Th2 immune responses.

The results of the prophylactic tumor assay showed that nsGO/PCP/OVA significantly inhibited tumor growth, suggesting the superior capability of nsGO/PCP/OVA to prevent tumor occurrence. The production of IgG antibodies in the nanoparticle groups further confirmed the ability of nsGO/PCP/OVA to induce strong humoral immune responses. As nsGO/PCP/OVA vaccination could effectively inhibit tumor growth after prophylactic immunization, we sought to investigate nanoparticle-mediated adaptive immune responses in therapeutic vaccinations. Similar to the prophylactic vaccination, tumors generated in nsGO/PCP/OVA-immunized mice exhibited a tendency of suppression compared to that in the PBS- or OVA + PCP-immunized groups. In addition, the production of IL-4 and IgG antibodies in nanoparticle groups were significantly increased compared to other groups. A recent study showed that GO can induce the differentiation of Th0 cells into Th2 cells, resulting in the promotion of humoral immunity (Lategan et al., 2018). Thus, our results further confirmed the hypothesis that tumor antigen-specific CD4^+^ T cells are functionally activated by nsGO/PCP/OVA nanoparticle and exhibit strong humoral immune responses.

In addition, our research concerning nanoparticle vaccines leaves some room for improvement. In subsequent research, it will be necessary to investigate immunological memory induced by nanoparticle vaccines in surviving mice as a supplement (Chen et al., 2016; Yang et al., 2017). Moreover, several reports have shown that cancer vaccines can be combined with immune checkpoint blockade (ICB) therapies, such as α-PD-1, to improve therapeutic outcomes (Egen et al., 2002; Topalian et al., 2012; Crittenden et al., 2015; Melero et al., 2015). Hence, further research should be undertaken to compare the therapeutic efficacy of nsGO/PCP/OVA nanovaccines and nanovaccines in combination with α-PD-1 therapy against established tumors. Although nsGO vaccine technology has shown robust immune effects, its relatively poor stability and low solubility limit its application in the human body (Zheng et al., 2018). Therefore, further modifications of nsGO are required to achieve improved biocompatibility.

## Conclusion

In this study, nsGO/PCP/OVA nanoparticles were constructed for tumor immunotherapy by encapsulating the antigen protein OVA and adjuvant PCP with nsGO nanosheets. Nanoparticles enhance the cellular uptake of antigens, promote the maturation of BMDCs *in vitro*, and induce both Th1 and Th2 responses *in vivo*. Importantly, nsGO/PCP/OVA could be utilized not only as a prophylactic nanovaccine against E.G7-OVA tumor challenge, but also as a therapeutic nanovaccine to inhibit the growth of existing tumors. Given the urgent need for safe vaccination platforms that trigger humoral and cellular immunity in the treatment of malignancies, the nanoparticle vaccine described here is of potential value for future applications in cancer treatment.

## Data Availability

The original contributions presented in the study are included in the article/Supplementary Material, further inquiries can be directed to the corresponding authors.
