# Supplementary figures and images for: Poria cocos polysaccharide—functionalized graphene oxide nanosheet induces efficient cancer immunotherapy in mice
Source: Front Bioeng Biotechnol. 2023 Jan 16;10:1050077. doi: 10.3389/fbioe.2022.1050077 (PMC9885324; doi:10.3389/fbioe.2022.1050077)

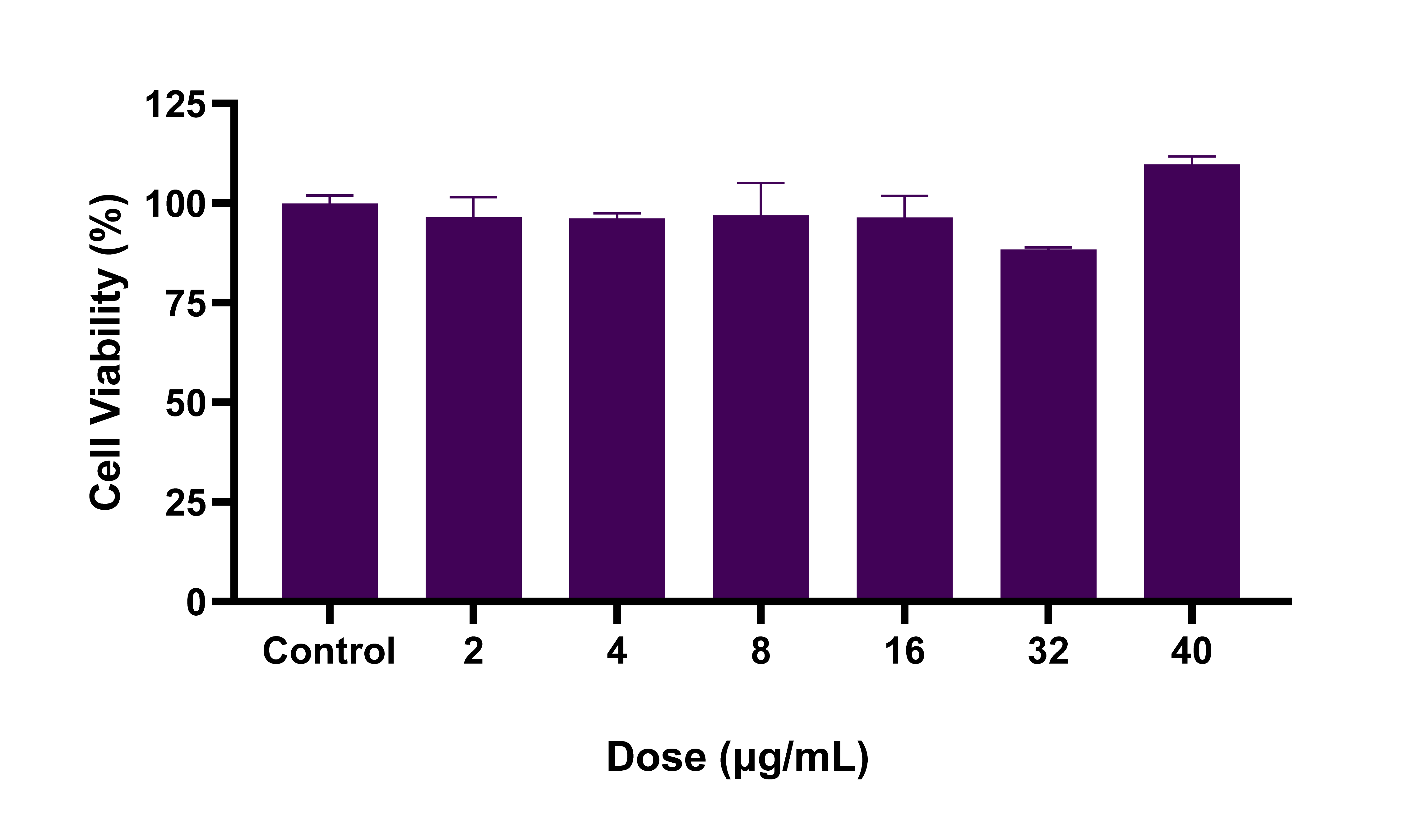

Supplement: Supplementary file 1 [file Image2.tif]

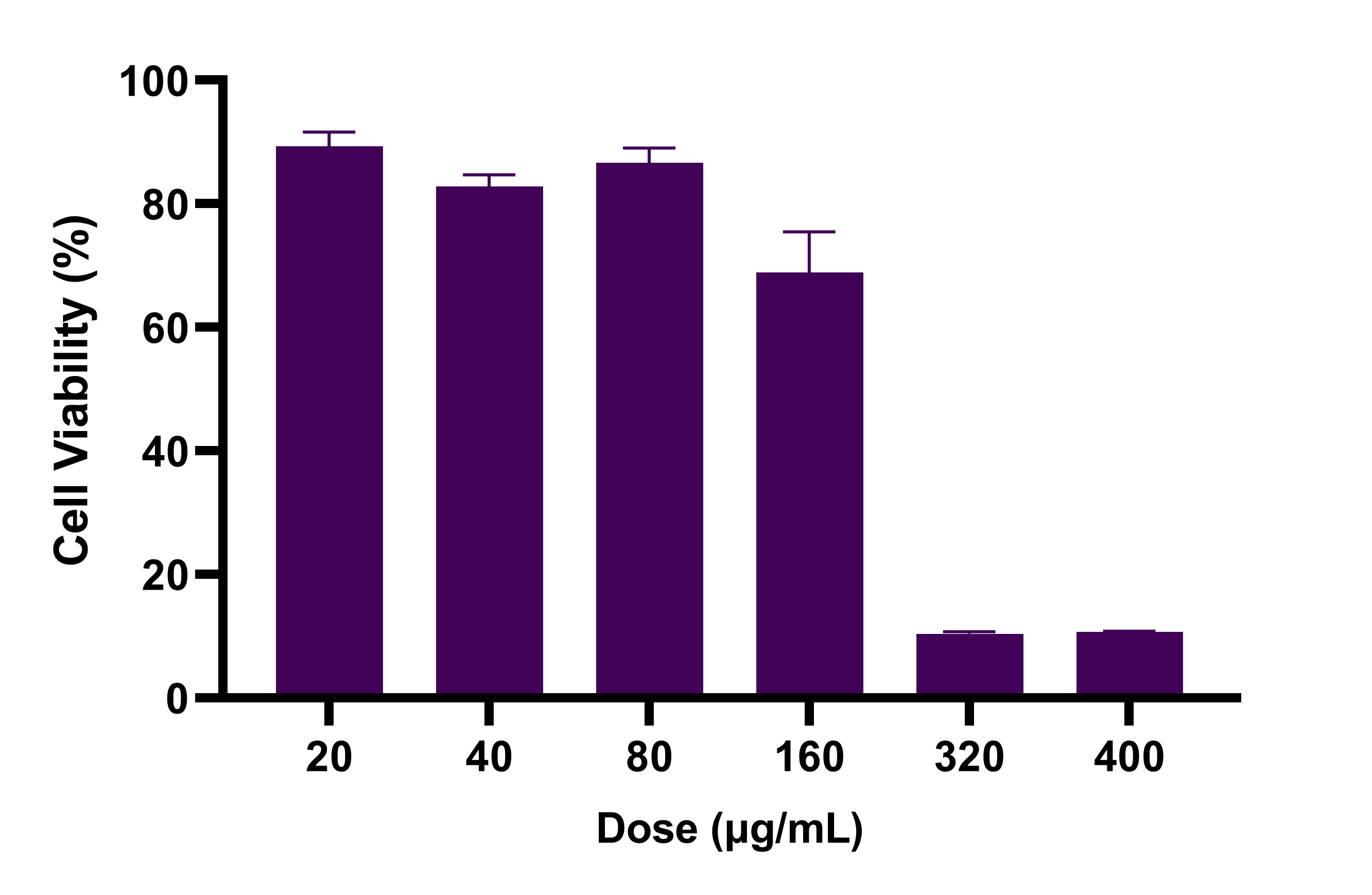

Supplement: Supplementary file 2 [file Image1.tif]
