# Supplementary material for: Poria cocos polysaccharide—functionalized graphene oxide nanosheet induces efficient cancer immunotherapy in mice
Source: Front Bioeng Biotechnol. 2023 Jan 16;10:1050077. doi: 10.3389/fbioe.2022.1050077 (PMC9885324; doi:10.3389/fbioe.2022.1050077)
Supplement: Supplementary file 3 [file Image3.pdf]

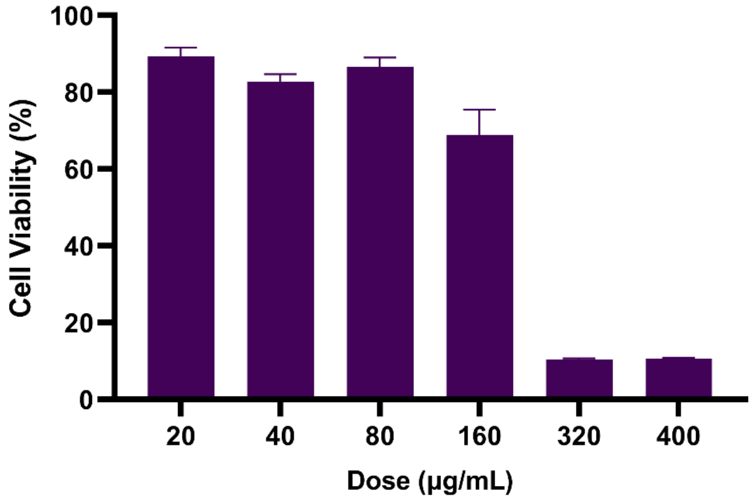

Fig. S1. (A) Cell viability of BMDCs after nsGO treatment was evaluated by CCK-8 assay.

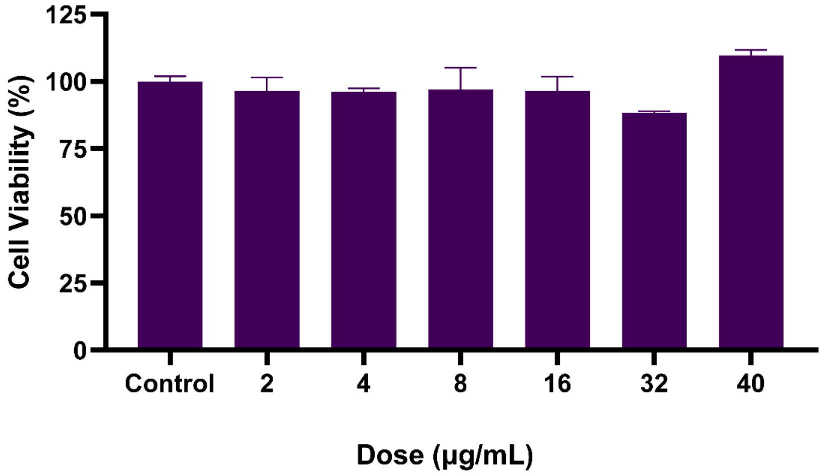

Fig. S2. (A) Cell viability of BMDCs after nsGO/PCP/OVA treatment was evaluated by CCK-8 assay.
